# The Persistence of High Levels of Living Alone Among Adults with Disabilities in Sweden, 1993–2011

**DOI:** 10.1007/s11113-020-09570-2

**Published:** 2020-02-05

**Authors:** Glenn Sandström, Fredinah Namatovu, Jens Ineland, Daniel Larsson, Nawi Ng, Mikael Stattin

**Affiliations:** 1 Centre for Demographic and Ageing Research, Umeå University, Umeå, Sweden; 2 Stockholm University Demography Unit (SUDA), Stockholm University, Stockholm, Sweden; 3 Department of Historical, Philosophical and Religious Studies, Umeå University, Umeå, Sweden; 4 Department of Education, Umeå University, Umeå, Sweden; 5 Department of Sociology, Umeå University, Umeå, Sweden; 6 Department of Epidemiology and Global Health, Umeå University, Umeå, Sweden

**Keywords:** Sweden, Disability, Living arrangements, One-person households, Disability legislation

## Abstract

This study investigates how the probability to live alone has developed among working age individuals with and without disabilities in Sweden during the period 1993–2011 when extensive political reforms to improve the integration of disabled individuals in society were implemented. The results show that individuals with disabilities are approximately twice as likely to be living alone when compared to individuals without disabilities. People with disabilities were also more likely to report low life satisfaction, and this was especially true among individuals with disabilities living alone. Men and women with disabilities also tend to experience longer periods of living as a one-person household than non-disabled people. Over time we find no indications of reduced differences in family outcomes between disabled and non-disabled individuals but rather evidence to the contrary. These differences are interpreted as being the result of the disadvantage disabled individual’s experience in the partner market and that people with disabilities are less successful in forming partnerships that can lead to cohabitation and family formation. The results thus show how disabled individuals still face societal barriers that limit their possibilities to find and sustain relationships that result in stable cohabitation despite increased efforts to improve their inclusion in Swedish society.

## Introduction

Among adults, living arrangements such as living alone, with kin or in a conjugal relationship are primarily the result of different individuals’ ability and preferences for family formation and the ability to sustain oneself in an independent household. The possibility to exit the parental home into either intendent living or cohabitation/marriage among young people is, however, a choice that is constrained both by socioeconomic and by health-related resources where disabled people experience stronger constraints than people without disabilities (Clarke and McKay 2014). Existing studies on family structure and union formation from a number of Western countries find a strong negative association between disability status and the probability of entering marriage or cohabitation (Liu and Zhang 2013; MacInnes 2011; Savage and McConnell 2016; Tumin 2016). Additionally, studies concerning disability’s impact on family dynamics consistently find a lower probability of becoming a parent among people with disabilities (Clarke and McKay 2014; Franklin 1977; Olsen and Clarke 2003; Morris and Wates 2006). A number of studies find that people with disabilities also are more likely to experience separation and divorce (Clarke and McKay 2014; Savage and McConnell 2016; Singleton 2012). However, at present, we do not know to what extent processes in the partner market and other potential mechanisms works to produce different living arrangements among people with disabilities in Sweden than those found in the general population. In Sweden, there have been very few studies on the impact of disability on different aspects of family behavior and even fewer regarding related issues such as the living arrangements of people with disabilities. The few studies that exist are focused on the subjective experience of people with disabilities living in institutional arrangements, as opposed to those living independently (Paulsson and Ringsby Jansson 2008; Häll and Skjöld 2003).

The aims of this study are as follows: (a) to investigate how disability is associated with the probability of living alone in Sweden; (b) to show to what extent this has changed in recent decades, which have been marked by extensive political reforms and the introduction of disability rights legislation in Sweden and; (c) to investigate whether there are differences in the subjective quality of life rating among those that live alone versus those that cohabit, for people with and without disabilities. The reason for focusing on how disability is associated with living alone is because singlehood is an indicator of an adult’s access to various forms of social support and his/her possibilities of resource and risk pooling.

## Background and Rationale

We know from previous research that living alone is associated with negative health outcomes and less access to social support. Scholars have reported that married people have better physical health, psychological well-being, and lower mortality compared to individuals that are single, divorced, separated, or widowed (Carr and Springer 2010; Chung and Kim 2014; Koskinen et al. 2007; Ross et al. 1990; Simon 2014). The protective effect of marriage on health and well-being is in part explained by the emotional and economic support provided by the partner (Dafoe and Colella 2016; Ross et al. 1990). Entering old age as a single individual can be a disadvantage, in terms of both health and economic resources, as there is no partner present that one can pool resources with (Tamborini 2007), nor the social support inherent in a marriage (Koball et al. 2010). Among people with disabilities, marital status is one of the most significant predictors of satisfaction with life, together with factors such as financial status, self-esteem, and health status (Kinney and Coyle 1992).

There have been several attempts to establish a link between disability and singlehood. The theory of assortative mating/homogamy has been used to explain the high prevalence of singlehood among people with disabilities (e.g., Tumin 2016). This theory suggests that high-resource individuals tend to partner with individuals having a similar status and resources. In recent decades, there is also a growing tendency for couples with high levels of education and income to exhibit lower levels of separation and divorce, when compared to individuals with low socioeconomic status. This tendency has increased in Scandinavian countries, as indicated by a shift to a positive socio-economic gradient in fertility and marriage, and a negative gradient with divorce/separation (Goldscheider et al. 2015; Esping-Andersen and Billari 2015). In disability research, these kinds of assortative mechanisms in the partner market have been characterized as “Disablist beliefs,” meaning people without disabilities discard people with disabilities as potential partners (Savage and McConnell 2016; Crawford and Ostrove 2003; Kalliantes and Rubenfeld 1997; O’Toole 2002; Robillard and Fichten 1983). Another mechanism proposed as an explanation for the positive association between disability and living alone is opportunity constraints, i.e., limited social participation, which reduces the possibilities of meeting potential partners. Studies from Great Britain show that only 29% of those aged 15–17 with physical disabilities had been on a date, as compared to 75% of adolescents without disabilities (Anderson et al. 2002). In Sweden, qualitative studies on women with disabilities from different generations found that disability results in strong constraints to finding a sexual partner, especially one that is not themselves disabled (Helmius 1999). Moreover, people without disabilities often fear being potentially caught up in a constraining caregiving role when entering a relationship with a partner with disabilities (Savage and McConnell 2016; Fiduccia 2000; Gill 1996).

Previous studies on the family status of people with disabilities have interpreted higher levels of divorce, singlehood, and childlessness among them as indications of social exclusion, rather than being a result of differences in preferences between people with and without disabilities (Franklin 1977; Singleton 2012; Clarke and McKay 2014; Savage and McConnell 2016; Tumin 2016). This assumption is supported by a number of studies that have failed to find any significant differences in preferences regarding family formation and cohabitation among people with and without disabilities, leading to the conclusion that differences in family outcomes can be primarily explained by the lack of social integration, and an inability for people with disabilities to participate in the partner market on an equal footing with people without disabilities (Arnold and Chapman 1992; Emerson et al. 2008; Nosek et al. 2001).

An important aspect about exploring the relationship between disability and the probability of living alone in Sweden is to address how ‘disability’ should be understood and defined. Previous research show that disability has been operationalized in different ways, which means that disability has been given different and sometimes contradictory meanings. Grönvik (2009) differentiates between three commonly held definitions: first, *functional limitations*, which stems from a medical understanding (disability as, e.g., blindness, deafness or other changes in bodily structures); second, *administrative definitions*, which originates from the distribution of welfare benefits, to decide who are and are not eligible for support, current legislation often define what is considered to be a disability in relation to a certain benefit; and third, a *subjective definition* of disability means that a person conceives of him- and herself as disabled; this means that inclusion in the category ‘disabled people’ is voluntarily (pp. 2–3). Grönvik (2009) indicates that a challenge with measuring disability is that different measures count different groups in the population. This is because, for example, the subjective definition of disability (i.e., whether or not a person considers oneself as having a disability) does not necessarily coincide with the administrative definition of disability (i.e., whether or not a person is assessed as 'having a disability' and consequentially being eligible for certain benefits).

In our analysis, we utilize two different types of indicators for disability status: first, a register-based definition including people with physical disabilities and mental disorders recognized by the Swedish Social Insurance Agency, which may be seen as an administrative definition; second, our analysis also includes people who subjectively have reported to be disabled (mobility disabilities), which relates to the subjective definition of disabilities. The use of both administrative and subjective/functional indicators is a strength of the data used in this analysis as it allows us to discern possible differences of these two indicators on our outcome in terms of how disability impacts the living arrangements of individuals.

## Disability Rights Legislation in Sweden

Given that family outcomes can be regarded as an indicator of social inclusion for people with disabilities, the continuity or change in the family behavior of this group is of interest in light of the extensive policy efforts undertaken in Sweden over recent decades to increase the inclusion of people with disabilities in society. Since the 1960s, the concept of normalization has been a key ideal and “conceptual banner” (Tössebro 2016, p. 112) in Swedish disability policy, setting the stage for deinstitutionalization and dedifferentiation in service provision (Ineland 2016). To ensure that all citizens have access to the same level and quality of education, services, and medical care, the state has certain responsibilities, such as enacting social policies, laws, and general welfare policies. Sweden’s disability policy is influenced by the United Nations Convention on the Rights of Persons with Disabilities, CRPD (United Nations 2007). CRPD was ratified by Sweden in 2008, and it obligates states to ensure access to a range of support services, including the personal assistance necessary to support living and inclusion in the community, and to prevent isolation or segregation from the community (ibid.). In recent years, disability policy has strongly emphasized active citizenship and how public policy through redistributive and regulatory measures enables citizens with disabilities to maintain security through social rights, personal autonomy, and influence in public deliberation and decision-making processes (Halvorsen et al. 2017; Sépulchre 2018). These developments are partly a response to citizens’ demands for increased self-determination and greater autonomy over decision-making in the community, rather than relying on state-based service provision (Ineland et al. 2019; van Toorn and Soldatic 2015).

A central pillar for supporting disabled people in Sweden includes ‘attendance allowance,’ regulated in The Social Insurance Code (2010, p. 110), which is a compensation to cover the cost for personal assistance to people with severe disabilities. However, in our analysis, we do not focus on the individuals receiving attendance allowance as our data do not allow us to distinguish them from other individuals having disabilities. Another important part of the public support system in Sweden for people with disabilities is the disability benefit program (Socialförsäkringsbalken 2010, p. 110. Kap 33). This social security scheme is a bit narrower than attendance allowance, since it provides income support only to working age persons with long-term limitations in their working capacity due to ill health. Work disability is defined in relation to incapacity to perform normal work tasks (Jönsson et al. 2010). With the aim of reducing the annual inflow to the disability benefit rolls, the eligibility criteria have been continuously tightened over the past decades (Johansson et al. 2018). Today, only medical reasons are recognized as grounds for granting benefits and the incapacity must be expected to endure for the foreseeable future. Most diagnoses are eligible grounds for disability benefit but there are three main diagnose groups that dominates in the stock of recipients. These are diseases of musculoskeletal system, mental and behavioral disorders, and diseases of the circulatory system. Until 2005 musculoskeletal diseases was the single most common cause of disability benefit. But since then mental disorders have become the largest diagnose group (Försäkringskasssan 2019). The pattern of diagnoses has thus changed considerably which is important to note when studying disability benefit recipients over time.

Given the increasingly narrowing eligibility for disability benefits, the inflow into the program has decreased. During the 1990s, between 40- and 60,000 people were annually granted disability benefits. Most of these received full benefits, under the assumption that they would be unable to return to the labor market. Today, that figure has decreased to 10–15,000 (Försäkringskassan 2018).

The main argument from policymakers for reforming the disability benefit program and tightening eligibility has been that receiving disability benefits is often a one-way trip; in other words, very few recipients leave the program and return to active work (Marin 2003; Johansson et al. 2018). Previously, unemployment and other social reasons in combination with less serious health issues were recognized as grounds for granting disability benefits, which means that the program has contributed to the permanent exclusion from work life of working age people with less severe illnesses. (Marin 2003; OECD 2003). This was not in accord with the general disability policy ambitions. However, one obvious consequence of the increasingly stricter eligibility for disability benefits is that recipients as a group over time typically have worse health.

It is therefore clear that disability policies in Sweden have changed quite substantially over recent decades. This has been done mainly in the direction of encouraging active citizenship, social inclusion, and personal autonomy, aiming especially at increasing the possibilities of, and incentives for, people with disabilities to be active in the labor market. However, it is unclear if the family outcomes of people with disabilities, such as the propensity to live alone, have changed in any significant way during this period, since the 1990s, of extensive change to disability rights policy.

## Data and Methods

To assess how disability is associated with the probability of living alone in Sweden, and to what extent the new rights legislation, and improvements in support and personal assistance, are associated with any changes in the share of people with disabilities that live alone, we used the Survey of Living Conditions (ULF/SILC), linked to register data from the Longitudinal Integration Database for Labor Market Studies (Statistics Sweden 2016). This was used to get register-based information on socio-economic status, geographical context, and information on whether an individual received disability benefits.

The ULF/SILC is conducted annually by Statistics Sweden, on behalf of Sweden’s parliament, since the late 1970s. The panel has a cross-sectional and a longitudinal part. The survey covers several welfare areas, such as income, health, marital and family status, accommodation, employment, and safety. The survey also includes in-depth modules (such as economy, labor market, health) implemented during different data collection waves and repeated every eighth year. We use the self-reported information answering the question if the person lives alone or not provided in the ULF survey, coded as a simple dichotomous variable. We choose to use the survey information rather than information on living arrangements available in the register of Statistics Sweden because information on household composition that correctly identifies cohabiting unmarried individuals that do not have shared biological children was not available in the registers for non-census years until 2011, when a dwelling register was introduced in Sweden. A discussion of these limitations in the register data is provided by Statistics Sweden (2003).

An additional benefit of using the ULF survey is that information on disability status in the registers of Statistics Sweden is limited to whether the individual receives disability benefits. Although we find that receiving disability benefits is a good indicator for all-cause disability, using the ULF/SILC survey has the advantage of enabling us to use self-reported indicators of disability status that can be contrasted to the one provided by the external assessment of disability status given by the social insurance agency, which to some extent is affected by changes in legislation and implementation of the laws. Lastly, using the ULF/SILC also provides additional information that we could not get from the register data, such as the individual assessment of quality of life, how long the individual has been living alone, social support, and so on which we can use to further contrast the living conditions of people with and without disabilities.

There are 17,241 observations in the working data, consisting of individuals aged between 25 and 64 at the time of interview. These individuals participated in the ULF/SILC 1993–1996 (wave 1), 2002–2003 (wave 2), or 2010–2011 (wave 3). This study included 11,580 unique individuals, as there were some who had participated in more than one wave of data collection. We use the data as cross-sections of the population at the time of interview and do not consider the panel element in the data in terms of analyzing any changes within the subjects over time. However, we do account for any potential clustering within subjects due to repeated measurements in our statistical analysis.

The time of the first interview, i.e., 1993–1996, is used as the baseline. This period coincides with the extensive expansion of public support to people with disabilities, in particular the introduction of the attendance allowance law providing the right to personal assistance for individuals with disabilities. We expect that any effects of these reforms will occur with some lag and may occur throughout the period of 1994–2011 (Government Board of Health and Welfare 2015, p. 17). Also, it is important to notice that the expansion of public support for people with disabilities does not concern disability benefit eligibility during the period. Disability benefit regulation has become stricter over time, as mentioned in previous paragraphs.

We use logistic regression to estimate the probability of being in a one-person household, depending on the disability status, while controlling for other demographic and socio-economic background characteristics. We code the living arrangement into a dichotomous variable of living alone vs. cohabiting with others and adjust for any within subject correlation across observations for the panel individuals by applying a clustered sandwich estimator of the standard errors. We also test an alternative multi-level specification in terms of random intercept models that control for within subject unobserved heterogeneity. However, these models did not yield any substantive differences in the estimated probabilities, and therefore, we choose, for reasons of parsimony, to present the simpler specification using a clustered sandwich estimator of the standard errors.

Differences in predicted probabilities between individuals having different combinations on covariate values are consistently reported in the form of the average marginal effect derived from the model estimates. For a discussion on different options available to calculate predicted probabilities from nonlinear probability models such as logistic regression see, e.g., Cameron and Trivedi (2009).

We used the linked data from the Longitudinal Integration Database for Labor Market Studies (Statistics Sweden 2016) to construct two different disability benefit indicators. The first is a dichotomous variable coded equal to one if the individual received any disability benefit, and zero otherwise. The social insurance agency [Försäkringskassan] may offer disability benefits on less than a full level, depending on one’s employment capacity. For individuals with less severe functional impairments that are still able to work some hours, the insurance agency is inclined to grant only part-time as opposed to full-time disability benefits. Therefore, we used the share of total disposable income received from disability benefits as a proxy indicator for the severity of the disability. We assume that individuals receiving a smaller fraction of their income from disability benefits have less severe functional impairments, on average. We tried a couple of specifications and found that an indicator with three levels differentiating between no disability benefit, less than 40%, and more than 40% of total income from disability benefits produced the best fitting model.

To contrast against the register-based definition of all-cause disability in terms of receiving disability benefits, which is based on the external evaluation of the social insurance agency, we also use the self-reported indicator for mobility impairment that is available in the ULF/SILC. We defined people with mobility impairment as those who responded “No” to the question “Can you run a distance of 100 meters if you are in a hurry?” and “No” to at least one of the additional questions: “Can you get on and off a bus (without assistance)?” or “Can you take a short walk of 5-minutes at a fairly rapid pace?” These definitions capture groups that to some extent are different but they also overlap. Running the two categories against one another shows that a majority of respondents reporting moving impairments also receive disability benefits due to musculoskeletal disorders. The disability benefit group on the other hand contains a much broader spectrum of ill health assessed by medical doctors which means that of health-related functional impairments at a trivial level are sorted out.

To control for socioeconomic differences, we include both levels of education and disposable income after taxation, including gains and losses from dividends in the year of interview, as defined in the LISA register. Disposable income was divided into percentiles based on the distribution of incomes for those in the age range of 25–64 during the year in question. To control for contextual differences, we include the type of municipality that the individual resides in at the time of the interview, based on the classification provided by the Association of Swedish Municipalities (2016). We use the highest aggregation level of (i) big cities of at least 200,000 inhabitants in the municipality, which corresponds to Sweden's three largest cities: Stockholm, Gothenburg, Malmö, and the surrounding municipalities that share the same labor market; (ii) medium size towns with less than 200,000, down to 40,000 inhabitants; and (iii) smaller towns with less than 40,000 inhabitants, including rural municipalities that are sparsely populated.

## Results

Descriptive statistics for the disability indicators, as well as all of the demographic and socio-economic variables included in the analysis for each period of observation, are presented in Table 1. A total of 4712 observations were included for the period of 1993–1996, 7574 observations for the period of 2002–2003, and 4955 observations for the period of 2010–2011. Less than 20% of the individuals reported that they lived alone at the time of the interview, about 3% of them reported a mobility impairment, and about 9% of them received disability benefits, according to the register data.

**Table 1:** Descriptive statistics

	ULF 1993–1996	ULF 2002–2003	ULF 2010–2011	Total
	(*N* = 4712)	(*N* = 7574)	(*N* = 4955)	(*N* = 17,241)
Person lives alone				
No	3899 (82.7%)	6158 (81.3%)	4065 (82.0%)	14,122 (81.9%)
Yes	813 (17.3%)	1416 (18.7%)	890 (18.0%)	3119 (18.1%)
Has mobility impairment				
No	4562 (96.8%)	7322 (96.7%)	4804 (97.0%)	16,688 (96.8%)
Yes	150 (3.2%)	252 (3.3%)	151 (3.0%)	553 (3.2%)
Receives disability benefits				
No	4304 (91.3%)	6873 (90.7%)	4581 (92.5%)	15,758 (91.4%)
Yes	408 (8.7%)	701 (9.3%)	374 (7.5%)	1483 (8.6%)
Share of disposable income from disability benefit				
0%	4304 (91.3%)	6873 (90.7%)	4581 (92.5%)	15,758 (91.4%)
–39%	154 (3.3%)	267 (3.5%)	154 (3.1%)	575 (3.3%)
40%–	254 (5.4%)	434 (5.7%)	220 (4.4%)	908 (5.3%)
Gender of respondent				
Woman	2417 (51.3%)	3873 (51.1%)	2597 (52.4%)	8887 (51.5%)
Men	2295 (48.7%)	3701 (48.9%)	2358 (47.6%)	8354 (48.5%)
5-year age group				
25–29	359 (7.6%)	936 (12.4%)	530 (10.7%)	1825 (10.6%)
30–34	446 (9.5%)	943 (12.5%)	594 (12.0%)	1983 (11.5%)
35–39	448 (9.5%)	901 (11.9%)	704 (14.2%)	2053 (11.9%)
40–44	463 (9.8%)	1020 (13.5%)	605 (12.2%)	2088 (12.1%)
45–49	806 (17.1%)	966 (12.8%)	686 (13.8%)	2458 (14.3%)
50–54	812 (17.2%)	987 (13.0%)	610 (12.3%)	2409 (14.0%)
55–59	731 (15.5%)	1036 13.7%)	512 (10.3%)	2279 (13.2%)
60–64	647 (13.7%)	785 (10.4%)	714 (14.4%)	2146 (12.4%)
Educational level (years)				
Primary (–9)	1132 (24.0%)	1044 (13.8%)	486 (9.8%)	2662 (15.4%)
Secondary (–12)	2153 (45.7%)	3547 (46.8%)	2223 (44.9%)	7923 (46.0%)
Undergraduate (> 15)	774 (16.4%)	1242 (16.4%)	834 (16.8%)	2850 (16.5%)
Graduate level (15–)	653 (13.9%)	1741 (23.0%)	1412 (28.5%)	3806 (22.1%)
Income percentile				
–20%	947 (20.1%)	1517 (20.0%)	983 (19.8%)	3447 (20.0%)
21–40%	941 (20.0%)	1518 (20.0%)	993 (20.0%)	3452 (20.0%)
41–60%	941 (20.0%)	1513 (20.0%)	993 (20.0%)	3447 (20.0%)
61–80%	944 (20.0%)	1517 (20.0%)	994 (20.1%)	3455 (20.0%)
81%–	939 (19.9%)	1509 (19.9%)	992 (20.0%)	3440 (20.0%)
Type of municipality				
Stockholm, Gothenburg, Malmö	1416 (30.1%)	2572 (34.0%)	1720 (34.7%)	5708 (33.1%)
City 40–200 K inhabit	1832 (38.9%)	2918 (38.5%)	1968 (39.7%)	6718 (39.0%)
Smaller towns and rural	1464 (31.1%)	2084 (27.5%)	1267 (25.6%)	4815 (27.9%)

*Source* Survey of living conditions ULF/SILC and Longitudinal Integration Database for Labor Market Studies (LISA) 1993–1996, 2002–2003, 2010–2011

Tables 2 and 3 show the living arrangements of individuals with and without disabilities, with disability status assessment based on either the presence of a mobility impairment (Table 2), or the receiving of disability benefits (Table 3) at the three survey periods. In Table 2, the proportion of individuals with a moving impairment who lived alone with no children was consistently higher than that of individuals without a mobility impairment, i.e., 27.3%, 27.0%, and 31.1% vs. 16.9%, 18.4%, and 17.5% in 1993–1996, 2002–2003, and 2010–2011, respectively. The proportion of individuals with a moving impairment who lived in a nuclear family with children was also relatively constant during the three surveys, ranging from 23.3 in 1993–1996 to 26.5% in 2010–2011, while the proportion among people without disabilities was almost twice as high, ranging from 42.4 to 44.6%. In general, there was no substantial increase in family formation among people with disabilities, but instead signs of the opposite, as the percentage of individuals living alone increased from 27.3 in 1993–96 to 31.1% in 2010–2011, and the percentage of individuals living with people that were not their parents, siblings, or children decreased from approximately 43% in 1993–1996 to 33% in 2010–2011. The persisting pattern over time was the much higher proportion of people with disabilities living alone, when compared to people without disabilities.

**Table 2:** Living arrangements by ULF-years and disability status, individuals aged 25–64, relative frequencies in percent

	Reports mobility impairment					
	ULF 1993–1996		ULF 2002–2003		ULF 2010–2011	
Household status	No	Yes	No	Yes	No	Yes
Living with parents and/or siblings	1.1	2.0	1.4	1.6	1.4	2.0
Nuclear family with no children	34.6	42.7	30.7	38.1	29.2	32.5
Nuclear family with children	42.4	23.3	42.2	24.6	44.6	26.5
Single with children	4.6	4.0	6.6	8.3	6.2	6.6
Living alone with no children	16.9	27.3	18.4	27.0	17.5	31.1
Other	0.3	0.7	0.6	0.4	1.0	1.3
Total	100	100	100	100	100	100

*Source* Survey of living conditions ULF/SILC and Longitudinal Integration Database for Labor Market Studies (LISA) 1993–1996, 2002–2003, 2010–2011

**Table 3:** Living arrangements by ULF-years and disability benefit reception, individuals aged 25–64, relative frequencies in percent

	Receives disability benefits					
	ULF 1993–1996		ULF 2002–2003		ULF 2010–2011	
Household status	No	Yes	No	Yes	No	Yes
Living with parents and/or siblings	1.1	2.0	1.4	1.6	1.4	1.9
Nuclear family with no children	33.3	51.2	29.9	41.8	28.0	44.7
Nuclear family with children	44.2	16.2	44.0	19.0	46.3	17.1
Single with children	4.7	3.4	6.7	6.3	6.3	5.3
Living alone with no children	16.4	26.7	17.5	30.5	16.9	31.0
Other	0.3	0.5	0.6	0.9	1.1	0.0
Total	100	100	100	100	100	100

*Source* Survey of living conditions ULF/SILC and Longitudinal Integration Database for Labor Market Studies (LISA) 1993–1996, 2002–2003, 2010–2011

Table 3 shows the living arrangements of individuals receiving disability benefits, compared to those who do not. Using this indicator, we observe quite similar patterns of living arrangements. Of those who received disability benefits in 1993–1996, over half (51.2%) reported living in a nuclear family with no children, 26.7% lived alone with no children, and 16.2% lived in a nuclear family with children. In contrast, the majority of individuals who did not receive disability benefits lived in a nuclear family with children (44.2%) during the same period, 33.3% lived in a nuclear family without children, and only 16.4% lived alone with no children. Over the two following decades, the proportion of individuals who lived in nuclear families without children decreased among those receiving disability benefits (from 51.2 to 44.7%), and those who did not (from 33.3 to 28.0%). The proportion of those living alone was quite stable among those who did not receive disability benefits, but among those who received, it increased moderately, from 26.7 in 1993–1996 to 31% in 2010–2011.

In sum, the descriptive findings do not indicate any increase in family formation among people with disabilities during the period under investigation, regardless of the indicator used. Rather, we observe some increase in the share of people with disabilities living alone during the period of 1993–2011 and a more pronounced decrease of those living with non-relatives and no children in the household.

Table 4 shows three logistic regression models for the probability of living alone, depending on the different indicators of disability status, while controlling for the demographic and socio-economic characteristics of individuals with and without disabilities during each survey wave. We include all significant interactions, as well as a test for possible changes in the effects of a disability over time through an interaction between the disability status and the survey period.

**Table 4:** Logistic regressions of probability of living in a one-person household by mobility impairment, disability benefit reception, and share of disposable income from disability benefits for individuals aged 25–64 in Sweden, 1993–2011

Variables	Model 1	Model 2	Model 3
Has mobility impairment			
No	(Base)		
Yes	1.87**		
Receives disability benefits			
No		(Base)	
Yes		2.05***	
Share of disposable income from disability benefits			
0%			(Base)
–39%			2.28***
40%–			2.04***
Gender of respondent			
Woman	(Base)	(Base)	(Base)
Men	6.64***	7.10***	7.50***
Survey year			
ULF 1993–1996	(Base)	(Base)	(Base)
ULF 2002–2003	1.06	1.02	1.02
ULF 2010–2011	1.02	0.99	0.99
5-year age group			
25–29	5.43***	5.77***	5.80***
30–34	2.41***	2.50***	2.49***
35–39	1.10	1.13	1.12
40–44	(Base)	(Base)	(Base)
45–49	1.79***	1.77***	1.75***
50–54	3.04***	2.95***	2.93***
55–59	4.75***	4.52***	4.51***
60–64	5.54***	4.92***	4.86***
Educational level (years)			
Primary (–9)	(Base)	(Base)	(Base)
Secondary (–12)	0.96	0.99	1.00
Undergraduate (> 15)	0.92	0.97	0.97
Graduate level (15–)	0.98	1.03	1.04
Income percentile			
–20%	(Base)	(Base)	(Base)
21–40%	1.13	1.21*	1.30**
41–60%	1.47***	1.65***	1.79***
61–80%	1.47***	1.66***	1.80***
81–%	1.38**	1.58***	1.71***
Type of municipality			
Stockholm, Gothenburg, Malmö	(Base)	(Base)	(Base)
City 40–200 K inhab	0.70***	0.68***	0.68***
Smaller towns and rural	0.67***	0.64***	0.65***
Survey year * Has mobility impairment			
ULF 2002–2003 * Yes	0.93		
ULF 2010–2011 * Yes	1.22		
Survey year * Receives disability benefits			
ULF 2002–2003 * Yes		1.15	
ULF 2010–2011 * Yes		1.24	
Survey year * Income percentage from disability benefits			
ULF 2002–2003 * –39%			0.65
ULF 2002–2003 * 40–100%			1.60**
ULF 2010–2011 * –39%			0.64
ULF 2010–2011 * 40–100%			1.86**
Male * Has mobility impairment			
Men * Yes	0.75		
Male * Receives disability benefits			
Men * Yes		0.73*	
Male * Income percentage from disability benefits			
Men * –39%			1.01
Men * 40–100%			0.57**
Male* 5-year age group			
Men * 25–29	0.45***	0.44***	0.43***
Men * 30–34	0.78	0.77	0.77
Men * 35–39	1.01	0.99	1.00
Men * 40–44	(base)	(base)	(base)
Men * 45–49	0.64*	0.65*	0.65*
Men * 50–54	0.39***	0.40***	0.40***
Men * 55–59	0.28***	0.29***	0.29***
Men * 60–64	0.21***	0.22***	0.22***
Male * Income percentile			
Men * 21–40%	0.58***	0.56***	0.53***
Men * 41–60%	0.41***	0.39***	0.36***
Men * 61–80%	0.30***	0.28***	0.26***
Men * 81–%	0.22***	0.20***	0.19***
Male * Type of municipality			
Men * City 40–200 K inhab	1.26*	1.29*	1.28*
Men * Smaller towns and rural	1.05	1.08	1.07
Constant	0.06***	0.05***	0.05***

*Source* Survey of living conditions ULF/SILC and Longitudinal Integration Database for Labor Market Studies (LISA) 1993–1996, 2002–2003, 2010–2011

*p < .05; **p < .01; ***p < .001

Using either a mobility impairment, reception of disability benefits, or the share of disposable income from disability benefits as indicators of disability, we observe a significantly higher probability of living alone among people with disabilities compared to people without disabilities, after controlling for potential confounders. The odds ratios for living alone are approximately twice as high for disabled people compared to people without disabilities, net of other socioeconomic and demographic factors.

The indicator using the share of disposable income from disability benefits shows a positive gradient, where those receiving more than 40% of their income from disability benefits have a higher probability of living alone when compared both to those who don’t receive disability benefits and to those receiving less than 40% of their income from disability benefits.

**Fig. 1:**
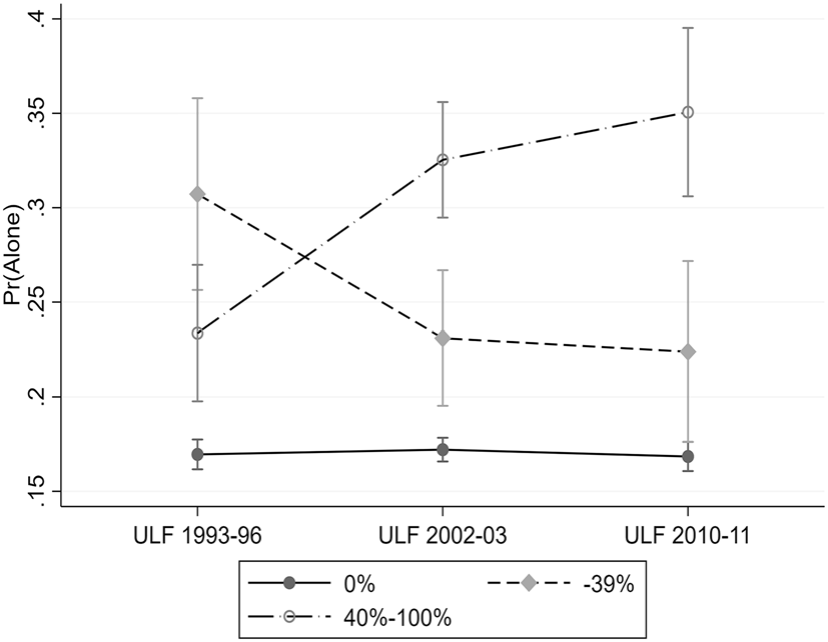
Probability of living alone for individuals aged 25–64, 1994–2011, by extent of disability benefit. *Source* Survey of living conditions ULF/SILC 1993–1996, 2002–2003, 2010–2011

However, this positive gradient is present only during the two latter survey periods of 2002–2003 and 2010–2011. This change over time of increasing singlehood among those receiving a higher share of their income from disability benefits is shown by the positive and significant interaction between the survey periods and those receiving more than 40% of their income from disability benefits. Figure 1 shows the estimated probabilities of living alone for different levels of disability benefit reception across the survey waves. Using this arguably more sensitive indicator that separate between those having different levels of total income from disability benefits provides evidence of an increased association between disability and living alone during the period of 1993–2011. In the first wave having disability benefits regardless of the extent significantly increases the probability to live alone. In the latter two waves, the “effect” of having more than 40% of income from disability benefits increases sharply and is significantly higher than in the first wave both in 2002–2003 and in 2010–2011. Additionally, in the latter two waves, a significant and positive gradient between having a higher or lower share of income from disability benefits is established. The significant positive effect of having part-time disability benefits as opposed to having none remains in the latter two waves if we are willing to accept a less conservative significance level of 0.1 for the last wave 2010–2011 where the contrast between having no income from disability benefits as opposed to having 1–39% increases the probability to live alone at a significance level of Sig. *P* < 0.071.

The results thus show a persistent positive association between all of our disability indicators and the probability of living alone for the period of 1993–2011. Additionally, the descriptive findings in Tables 2 and 3 showing increases in the share of people with disabilities living alone, and decreases in the level of cohabitation over time, are corroborated by our inferential analysis controlling for demographic and socioeconomic factors.

As for other variables included in the model, gender and age are by far the most influential factors. Men have approximately a 6.6–7.1 times higher adjusted odds ratio for living alone compared to women. However, the impact of disability on living alone is slightly lower among men than among women, as seen in the negative interaction effect of disability status for men, which is also significant in the case of men receiving disability benefits. Accounting for this interaction, the odds ratio for living alone among men receiving disability benefits is 1.50, compared to 2.05 for women after controlling for the other variables in Model 2. Yet, it is important to note that when comparing men and women with disabilities, disabled men are still more likely to live alone than disabled women, due to the strong association between being male and living in a one-person household. Using the estimates for the association between having disability benefits found in Model 2, Table 4, the estimated probability of living alone for men with disabilities is on average 31%, while it is only about 27% for women when controlling for the other variables in the model, although the influence of the disability status is lower for men than for women. Other groups with a higher probability of living alone are individuals younger than 35 and older than 45, men with low incomes, women in the higher income quintiles, and individuals living in large metropolitan areas, i.e., Stockholm, Gothenburg, and Malmö, when compared to their middle-aged counterparts, low-income women, and those who lived in cities, smaller town, and rural areas.

**Fig. 2:**
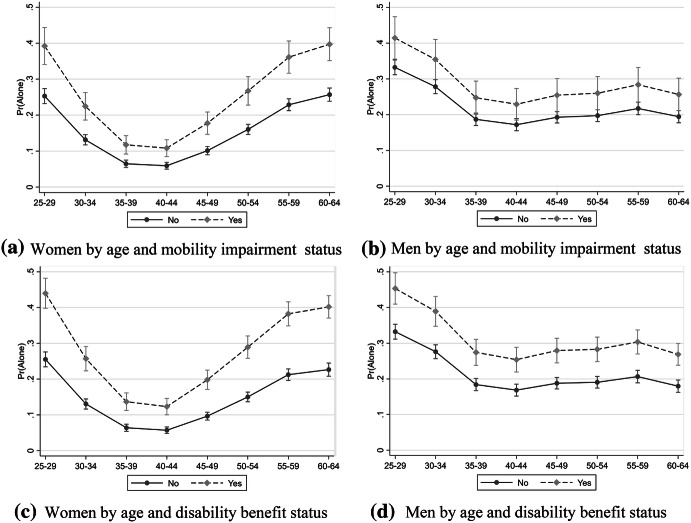
Probability of living in a one-person household for men and women aged 25–64, 1993–2011, by mobility impairment and disability benefit reception status. **a** Women by age and mobility impairment status, **b** Men by age and mobility impairment status, **c** Women by age and disability benefit status, **d** Men by age and disability benefit status. *Source* Survey of living conditions ULF/SILC and Longitudinal Integration Database for Labor Market Studies (LISA) 1993–1996, 2002–2003, 2010–2011

In Fig. 2, we show the estimated probabilities of living alone by age and disability status for men and women, respectively. From Fig. 2, it is clear that individuals with disabilities exhibit consistently higher probabilities for living alone compared to the general population without disabilities. Perhaps surprisingly, we find no indications of a difference for disabled and individuals without disabilities in the association between age and the probability of living alone. Rather, they follow the same age pattern as the population in general. Our sample shows the typical age pattern, where living alone is more common among the younger age group (before the stage of family formation), and in the age range of 45–65, when separation, divorce, and to a lesser extent mortality starts to contribute to union dissolution. These effects of age are more prominent among women than men.

### Differences in the Duration of Living Alone

**Fig. 3:**
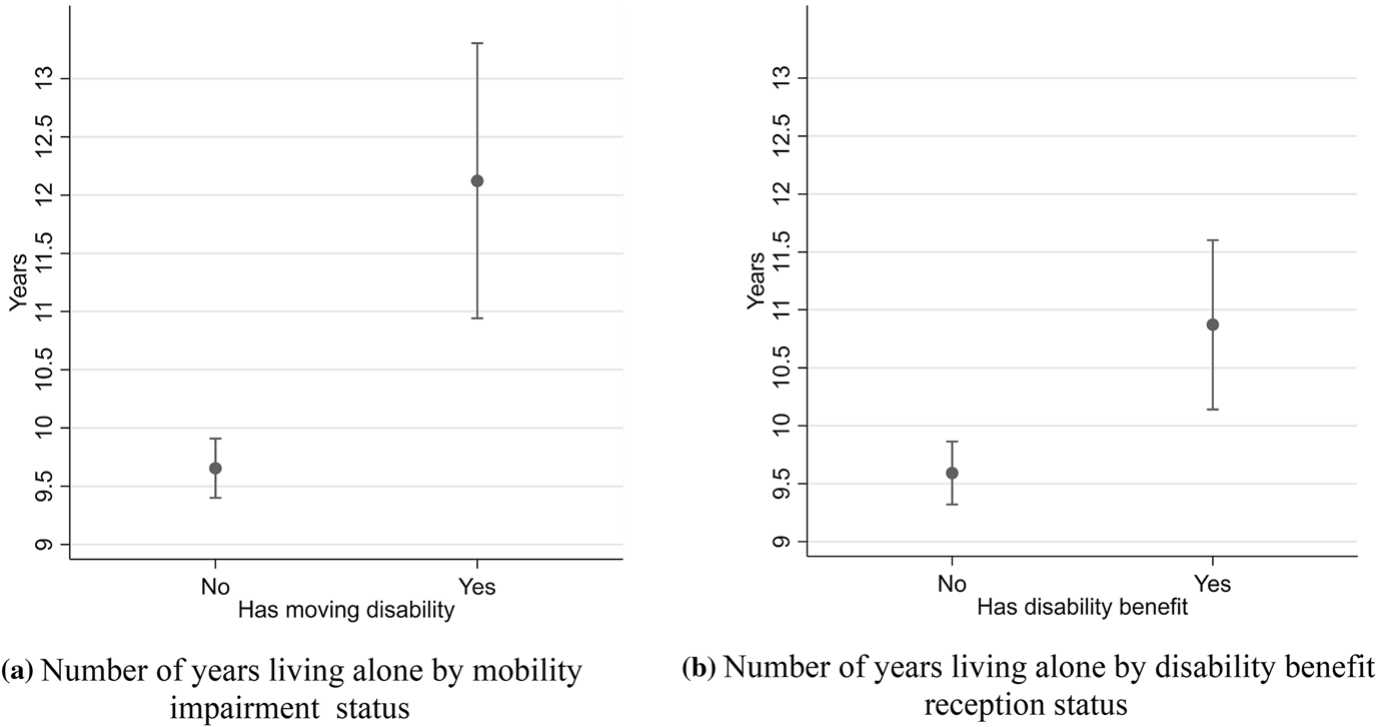
Mean number of years living in a one-person household, individuals aged 25–64, 1993–2003, by mobility impairment and disability benefit reception status. **a** Number of years living alone by mobility impairment status. **b** Number of years living alone by disability benefit reception status. *Source* Survey of living conditions ULF/SILC and Longitudinal Integration Database for Labor Market Studies (LISA) 1993–1996, 2002–2003. Measure is adjusted for age, sex, period, education, income, and municipality type

In the surveys conducted in 1993–1996 and 2002–2003, the respondents were asked how long they had lived alone if they lived in a one-person household at the time of interview. In Fig. 3, we show OLS regression estimates of the predicted mean number of years that the respondent stated that he/she had lived alone prior to the interview, depending on their disability status. Individuals with a moving impairment and those receiving disability benefits both reported having been in a single living arrangement for a significantly longer period of time when compared to individuals without disabilities who lived alone. We only show the dichotomous indicators in Fig. 3, as we found no evidence of longer periods of living alone for those receiving different levels of disability benefits. This was most likely due to the fact that we lack information for the period of 2010–2011, when the gradient between different levels of disability benefit uptake was the largest, according to our estimates in Table 4. The results show that people with disabilities living alone tend to have experienced longer periods of being in a one-person household and that there is likely an overrepresentation of individuals that have never cohabitated in the group having disabilities. Unfortunately, our data do not allow us to differentiate between these two alternative causes for the higher mean time of living alone among people with disabilities. It is probable, however, that both the longer durations and a higher proportion of never coupled individuals contribute to the association.

### Differences in the Subjective Quality of Life Rating

**Fig. 4:**
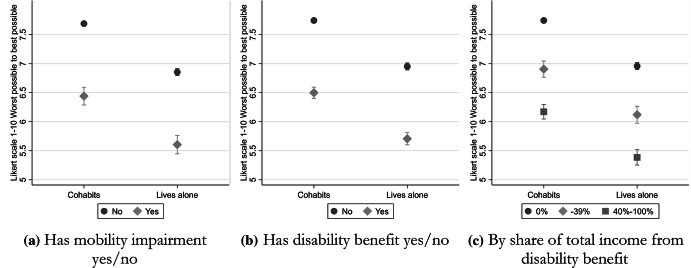
Subjective rating of quality of life from worst possible (1) to best possible (10) among individuals aged 25–64 living alone compared to cohabiting, 1994–2002, by disability status. **a** Has mobility impairment yes/no, **b** Has disability benefit yes/no, **c** By share of total income from disability benefit. *Source* Survey of living conditions ULF/SILC 1994–1996, 2002–2003, and Longitudinal Integration. Database for Labor Market Studies (LISA) 1993–1996, 2002–2003. Measure is adjusted for age, sex, period, education, income, and municipality type

In Fig. 4, we analyze the subjective quality of life rating among those living alone versus those that cohabit, according to disability status. In this case, the respondents were asked to rate their overall quality of life on a scale from 1 (worst possible) to 10 (best possible). This question was unfortunately also only available for the two first periods, 1993–1996 and 2002–2003. Individuals without disabilities who lived alone reported a statistically significant lower quality of life when compared to those who cohabit, as seen across the panels a-c in Fig. 4.

Individuals with disabilities, either measured based on a self-reported mobility impairment (Fig. 4, panel a), or disability benefit reception (Fig. 4, panel b), reported a substantially lower quality of life when compared to individuals without disabilities, and the contrast between those who lived alone and those who did not remained significant. In Fig. 4, panel c, we distinguish between those having a higher or lower share of their income from disability benefits, and here we see a clear gradient, where the individuals having more than 40% of their total income from disability benefits by far report the lowest overall quality of life ratings. The results indicate that both living alone and disability status are independently associated with the individual's tendency to report a lower quality of life, as there is no significant interaction between disability status and living arrangement. Taking both living arrangements and disability status into account, it is clear that people with disabilities who are living alone on average report a lower quality of life when compared to people without disabilities who cohabit.

## Concluding Discussion

The purpose of our study was to investigate whether disability is associated with a probability of living alone and to assess the extent to which this has changed over recent decades following disability rights legislation for the social inclusion of individuals with disabilities in Sweden. The results of this study suggest that people with disabilities aged 25–64 are significantly more likely to live alone when compared to people without disabilities. Furthermore, our results show that people with disabilities experience longer periods of living in a one-person household and that both their single status and disability status are associated with reporting a lower quality of life, when compared to people without disabilities and those that cohabit. This study found no evidence of an increase in family formation and parenthood among people with disabilities during the two decades covered by our analysis.

In general, men had significantly higher adjusted odds of living alone, but the increase in the probability of living alone for people with disabilities was slightly higher for women than for men. A potential mechanism that could influence the preferences for cohabitation among individuals with disabilities is if they stand the risk of losing social benefits if they enter marriage/cohabitation. Although we cannot completely rule out this type of causality between living arrangements and disability as the eligibility for housing benefits to some extent is influenced by cohabitational status, we argue that it is likely of less importance than other factors.

The reason for this conclusion is that eligibility for our indicator for all-cause disability is solely based on the capacity to participate in the labor market. Access to support in the form of services to adults with disabilities is also formally not dependent on the living arrangements of the individual in the Swedish system. But more importantly, the fact that the negative association between quality of life assessment and living alone is at least as large among disabled individuals as among individuals without disabilities indicates that it is unlikely that the higher proportions of living alone, and longer periods of living in a one-person household, primarily can be explained by a stronger preferences for solo living among individuals with disabilities than in the general population. Rather, it is likely that these patterns primarily reflect the disadvantages disabled individuals have in the partner market, and that people with disabilities are less successful in forming partnerships that can lead to cohabitation and family formation.

Additionally, the policy efforts implemented since the 1990s to integrate people with disabilities into wider society do not appear to have changed this disadvantage. The strong link between disability status and living alone revealed by this study is in line with the findings reported by earlier studies for other Western countries (MacInnes 2011; Savage and McConnell 2016; Tumin 2016) and suggests that people with disabilities have less opportunities to meet a potential partner. Some previous studies of family status among people with disabilities have tied the higher levels of singlehood and childlessness to social exclusion (Jamieson et al. 2009). This could be due to disablist beliefs, meaning that people without disabilities do not consider people with disabilities as potential partners (Savage and McConnell 2016; Crawford and Ostrove 2003). Swedish qualitative studies on women with disabilities find that disability results in strong constraints to finding a sexual partner, especially one that is not disabled (Helmius 1999). This conclusion is reinforced by the quantitative findings of this study, which show about twice as high levels of living alone among adults with disabilities compared to the those without disabilities, after controlling for other demographic, socioeconomic, and contextual indicators known to influence living arrangements. Several studies find that adolescents with disabilities have normative expectations; they expect and want to enter into cohabiting relationships and start a family of their own (Arnold and Chapman 1992; Bernert 2011). Internalization of negative messages received by people with disabilities during childhood concerning their potential to assume roles as partners or parents negatively impact their future views on partnership and parenthood, according to this research (Olsen and Clarke 2003; Sherry 2003). It is likely that the higher incidence of living alone found in this study among people with disabilities reflects the constraints working against the possibility of finding a suitable partner.

We report no significant changes in the levels of people with disabilities entering unions, either as cohabiting parents or in unions with no children, during the period of 1993–2011. We expected some decrease in the difference in union formation between people with disabilities and people without disabilities, considering that, in the 1990s, Sweden introduced reforms with extensive policies and support systems aimed at improving the participation of people with disabilities in society. The findings of this study indicate that people with disabilities experience persisting difficulties in navigating family dynamics and living arrangements, despite political reforms. A persistence of a high level of living alone among individuals with disabilities is possibly due to the fact that political reforms have mainly been implemented within the institutional framework of service provision focusing on living environment, occupation, and increased autonomy, and as such did not directly address family dynamics. Interestingly, a US study of the period of 1997–2013 notes that despite improvements in disability rights legislation and increased political activism advocating for the integration of people with disabilities in society, disparities in marriage rates continued to increase, rather than decrease, between people with disabilities and without disabilities (Tumin, 2016). Similar to these US findings, we find an increase in the association between living alone and having a higher share of total disposable income (> 40%) from disability benefits. However, we suggest that selection effects can be one possible explanation for the increased association. The tightening of eligibility criteria for receiving full disability benefits since the 1990s might work to increase the share of individuals with more severe functional impairments in the group receiving near full and full benefits. In turn, this might be one of the reasons for the increased association that we find from 2002 to 2003 and onwards.

Our study showed that men had significantly higher adjusted odds of living alone compared to women, although disability increased the probability of living alone slightly more for women than for men. However, the higher baseline risk among men compared to women means that, overall, men with disabilities are more likely to live alone than women with disabilities. This finding is in line with evidence from recent studies in Europe (Jamieson et al. 2009). In an attempt to explain the increase of men living alone, the role of men’s economic uncertainty in the postponement of marriage was highlighted (Oppenheimer 1988). This view is especially relevant in the context of men with disabilities living alone, due to the strongly negative effect of income on the probability of living alone among men. Being a recipient of disability benefits directly implies that the individual, at best, is only partially employed, which increases economic constraints. A UK study reported that subgroups of economically disadvantaged young men faced delays in transitions to partnership (Stone et al. 2011). Moreover, the probability of ending up in a one-person household after separation, rather than being left as a single parent, is likely higher for men than for women, which partially explains the higher rates of living alone among men with and without disabilities in the age groups when family formation is most prominent, between ages 35 and 45, during which women exhibit much lower probabilities of living alone. Nevertheless, singlehood among men with disabilities could have potential implications related to the male gender, and to disability. The observed reduced life satisfaction among those living alone, compared to coupled individuals, was at least as strong among people with disabilities as among those without disabilities. Disability and living alone might both lower life satisfaction because of other related constraints, including socio-economic disadvantages. Lower life satisfaction among people with disabilities raises important health questions, as life satisfaction is associated with beneficial health outcomes, including mental well-being (Bellis et al. 2012) and longevity (Collins et al. 2009; Wiest et al. 2011).

In conclusion, the results of this study show that working age adults with disabilities in Sweden are approximately twice as likely to be living alone when compared to individuals without disabilities. People with disabilities were also more likely to report low life satisfaction, and this was especially true among individuals with disabilities living alone. Although Sweden has worked extensively on social inclusion, and on reducing inequalities for people with disabilities, some of these differences still persist. As people with disabilities are more prone to social isolation, there is a need for further research to clarify the direct and indirect pathways leading to this association. If the ability to form and sustain family relationships is viewed as an important aspect of social inclusion, future research focusing on why policy appears to be unable to directly influence family outcomes among people with disabilities would be welcome.
